# Unravelling the *Helianthus tuberosus* L. (Jerusalem Artichoke, Kiku-Imo) Tuber Proteome by Label-Free Quantitative Proteomics

**DOI:** 10.3390/molecules27031111

**Published:** 2022-02-07

**Authors:** Ranjith Kumar Bakku, Ravi Gupta, Cheol-Woo Min, Sun-Tae Kim, Genboku Takahashi, Junko Shibato, Seiji Shioda, Fumiko Takenoya, Ganesh Kumar Agrawal, Randeep Rakwal

**Affiliations:** 1 Faculty of Engineering Information and Systems, University of Tsukuba, 1-1-1 Tenodai, Tsukuba 305-8572, Japan; ranjithkumar.bakku@gmail.com; 2 College of General Education, Kookmin University, Seoul 02707, Korea; ravigupta07@ymail.com; 3 Department of Plant Bioscience, Life and Industry Convergence Research Institute, Pusan National University, Miryang 50463, Korea; min0685@naver.com; 4 Zen-Yoga Institute, 3916 Okusa, Nakagawa-mura, Kamiina-gun, Nagano 399-3801, Japan; tgenboku@gmail.com; 5 Department of Functional Morphology, Shonan University Medical Sciences, 16-48 Kamishinano, Totsuka-ku, Yokohama 244-0806, Japan; rjunko@nifty.com (J.S.); seiji.shioda@sums.ac.jp (S.S.); 6 Department of Physiology and Molecular Sciences, Hoshi University, 4-41 Ebara 2-chome, Shinagawa, Tokyo 142-8501, Japan; kuki@hoshi.ac.jp; 7 Research Laboratory for Biotechnology and Biochemistry (RLABB), GPO 13265, Kathmandu 44600, Nepal; gkagrawal123@gmail.com; 8 Faculty of Health and Sport Sciences, University of Tsukuba, 1-1-1 Tennodai, Tsukuba 305-8574, Japan

**Keywords:** kiku-imo tuber, dried powder, health, human, plant, LC-MS/MS

## Abstract

The present research investigates the tuber proteome of the ‘medicinal’ plant *Jerusalem artichoke* (abbreviated as JA) (*Helianthus tuberosus* L.) using a high-throughput proteomics technique. Although JA has been historically known to the Native Americans, it was introduced to Europe in the late 19th century and later spread to Japan (referred to as ‘kiku-imo’) as a folk remedy for diabetes. Genboku Takahashi research group has been working on the cultivation and utilization of kiku-imo tuber as a traditional/alternative medicine in daily life and researched on the lowering of blood sugar level, HbA1c, etc., in human subjects (unpublished data). Understanding the protein components of the tuber may shed light on its healing properties, especially related to diabetes. Using three commercially processed JA tuber products (dried powder and dried chips) we performed total protein extraction on the powdered samples using a label-free quantitate proteomic approach (mass spectrometry) and catalogued for the first time a comprehensive protein list for the JA tuber. A total of 2967 protein groups were identified, statistically analyzed, and further categorized into different protein classes using bioinformatics techniques. We discussed the association of these proteins to health and disease regulatory metabolism. Data are available via ProteomeXchange with identifier PXD030744.

## 1. Introduction

**Figure 1:**
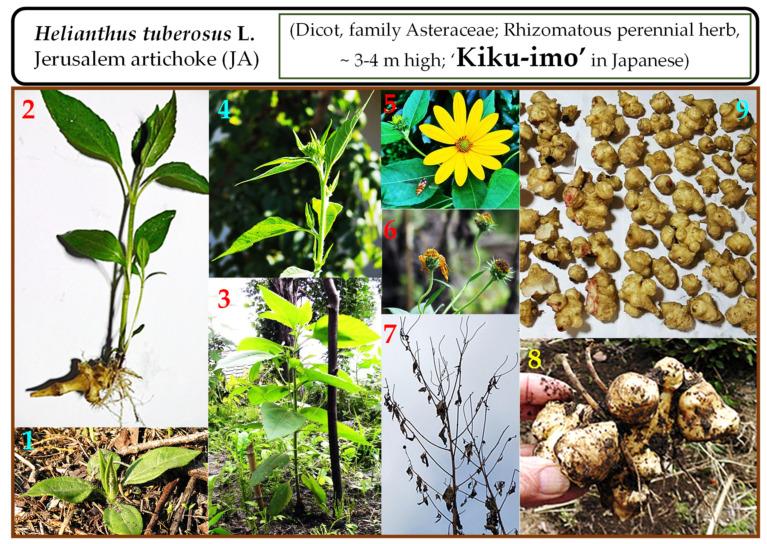
The *Helianthus tuberosus* L. (*Jerusalem artichoke*; JA, commonly called ‘Kiku-imo’ in Japanese) plant at different stages of growth: from sprouting till harvesting of the tubers. (**1**) Sprouting from the soil, (**2**) Sprout arising from the tuber, (**3**) Vegetative growth stage, (**4**) Flowering stage, (**5**) Flower, (**6**) Withered flower stage, (**7**) Wilting plant stage (is where the tubers are ready to be harvested), (**8**) Freshly dug tubers, and (**9**) Tubers.

*Helianthus tuberosus* L., commonly known as *Jerusalem artichoke* (abbreviated as JA), is an annual herb that belongs to the sunflower family, Asteraceae (Figure 1). This plant is well known for its resistance to environmental stress and thrives in diverse soil (arid-saline) and temperature (cold-hot) conditions [1,2,3,4]. It is cultivated in most parts of the world due to its diverse habitat and resistance to both biotic and abiotic stresses. In addition to its ability to thrive, the plant is also well known for its significance in feedstock, functional food, biomass, biomedical, and chemical industries [2,5,6,7]. A variety of phytochemicals such as phenols, flavonoids, coumarins, unsaturated fatty acids, polyacetylene derivatives, sesquiterpenes, protein, amino acid, reducing sugars, organic acids, lactones, cardiac glycoside, terpenoids, amino acids such as linoleic, α-linoleic acid, and inulin are produced in various parts (stems, leaves, flowers, and tubers) of the plant [8,9,10,11,12,13,14]. The plant species grown in Japan has been presented as a reference to understand its various growth stages from sprouting to tuber formation and harvesting (Figure 1).

Due to this diversity in functional-molecular components, the plant plays an important role in the health sector. More specifically, the areal parts of the plant are useful in pharmaceutical applications such as antioxidant, anticancer, antifungal, antidiabetic, antimicrobial, immune stimulation, etc., [13,15]. Although the tubers also provide such benefits through direct consumption (as food or supplements), they are more useful in promoting gut bacteria and treating chronic diseases such as diabetes [2,16,17]. Type 2 diabetes mellitus (DM2) is a common term for increased blood sugar level. It is a metabolic disorder characterized by hyperglycemic (lack of insulin) condition in the body. The condition is also associated with excessive loss of proteins, damage of several metabolic pathways and other disorders such as chronic kidney disease (CKD) and non-alcoholic fatty liver (NAFL) [18,19]. Most of these conditions are a result of unbalanced dietary habits that include an excessive intake of high-calorie and high-fat foods [20,21]. The use of plant extracts to decrease blood sugar level has been promoted since ancient times. However, it is only recently that the constituents of the plants and their role in human disease treatment are more keenly understood [22] through advanced omics approaches such as genomics, transcriptomics, proteomics, and metabolomics [23].

Several metabolomics and bio-chemical studies have been performed to unravel the chemical composition of the JA plant and its tubers [8,24]. However, very less is known about the proteome of the tubers. Plant proteomics provide significant insights into the biochemical pathways of essential metabolites, markers for disease treatment, as well as plant phenotype identification [25,26,27]. Most recent proteomic studies focused on understanding the salt stress responses [28] and molecular basis of carbohydrate metabolism [27] in JA. The latter provides more information on the biosynthesis mechanism of inulin (a linear polymer of D-fructose) in JA [27]. It is one of the major bioactive components produced by JA and highly abundant in its tubers. Inulin is known for promoting gut bacteria, improves glucose tolerance and liver lipid profile [2,29,30,31]. Apart from Inulin, other phytochemicals such as phenolics (cathecins, flavonoids, etc.) are also known to have significant roles in treating DM2 and related diseases such as chronic kidney disease (CKD) and Nonalcoholic Fatty Liver (NAFL) disease through their antioxidant properties [32,33,34,35,36,37,38,39,40,41,42,43,44]. Therefore, investigating the JA tuber proteome might provide new insights into the protein components and their healing properties. In particular, essential pathways related to bioactive components that are useful in promoting health including fighting life-style diseases such as DM2. Importantly, the proteome of a commercial or processed edible tuber source could help us to understand the significance of JA tubers as a functional food source.

With this background, and a first step in our research project, we decided to investigate the proteins present in the commercial edible samples (processed from tubers of the same plant species) that are available in Japan. The commonly available ready to eat (or use as additive in foods) products are first, the dried powder (as it can easily added into rice, or other food preparations) and second, dried chips that can be eaten as a healthy snack. It was also reasoned that the availability of their proteome profiles would give insight into not only common but unique proteins, which could be further used to explain the value of these products. Two different dried tuber powders (labeled as samples 1 and 2; see Supplementary Figure S1) prepared using slightly different drying techniques were used as a comparison. The powdered extracts of all three samples (1, 2, and 3; Supplementary Figure S1) were used to extract the total proteins that were analyzed by liquid chromatograph–tandem mass spectrometry (LC–MS/MS), and the highly significant proteins and pathways were identified through bioinformatics analyses.

## 2. Results

### 2.1. MS-Based Proteomics Approach Identifies the Tuber Proteome

**Figure 2:**
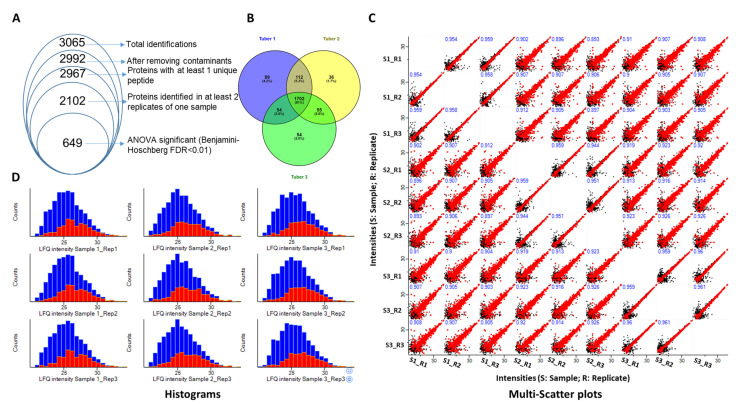
Significance of the proteomic changes among three (1, 2, and 3) different JA tuber samples. (**A**) Total detected protein groups in all three tuber samples while removing contaminants, identifying high-confidence protein groups in at least two samples, and performing the ANOVA test. (**B**) Venn diagram showing commonly and uniquely identified protein groups in all the three tuber samples. (**C**) Histograms showing LFQ intensity counts of the total (blue) and differentially modulated proteins (red). (**D**) Histograms show LFQ intensity counts of the total (blue) and differentially modulated proteins (red).

A label-free quantitate proteomic approach is used for analyzing the differential protein abundance among the three tuber samples. In total, 3065 protein groups are identified of which 73 are contaminants and were removed from the list. Another, 25 protein groups were identified without any unique peptide and were considered as low-confidence protein groups. After removing these low-confidence proteins, a total of 2967 high-confidence protein groups are identified (Figure 2). Considering a different processing of the tuber for tuber samples 1 and 2 (powdered samples) versus tuber sample 3 (sliced dried chips) the obtained protein data and categorization (despite the lack of a sequenced *H. tuberosus* L. genome, and therein a reason to use the *H. annus* database) commonly linked tuber 1 and 2 proteome closely compared with a slightly different tuber 3.

Because of the use of three different tuber samples, multiple missing values are expected after the MS runs; therefore, protein groups that were reproducibly identified in two of the three replicates (2102 protein groups) are selected and considered for further analysis (Figure 2, Supplementary Table S1). Table 1 shows the total number of proteins in each sample.

Venn diagram analysis showed 1702 protein groups which are commonly identified in all the three tuber samples, whereas 89, 36, and 54 protein groups are only identified in tubers 1, 2 and 3, respectively. Further, 112 protein groups were shared by tubers 1 and 2 indicating that their proteome is more closely related as compared with tuber pairs (2,3) and (3,1) (Figure 2A,B). To examine the reproducibility of label-free protein quantification among triplicates of the same sample, multi-scatter plots are generated using Perseus software (Figure 2C). Scatterplots of the same samples showed a typical non-uniform spread, that is, wider at lower intensities and pointed at higher. This is because of low accuracy and reproducibility for the peptides that are closer to the background level. Nevertheless, the Pearson correlation coefficient of different replicates of the same samples is more than 0.944, suggesting a high degree of correlation among triplicates of the same samples. Histograms show the protein counts before and after missing value imputation (Figure 2D).

**Figure 3:**
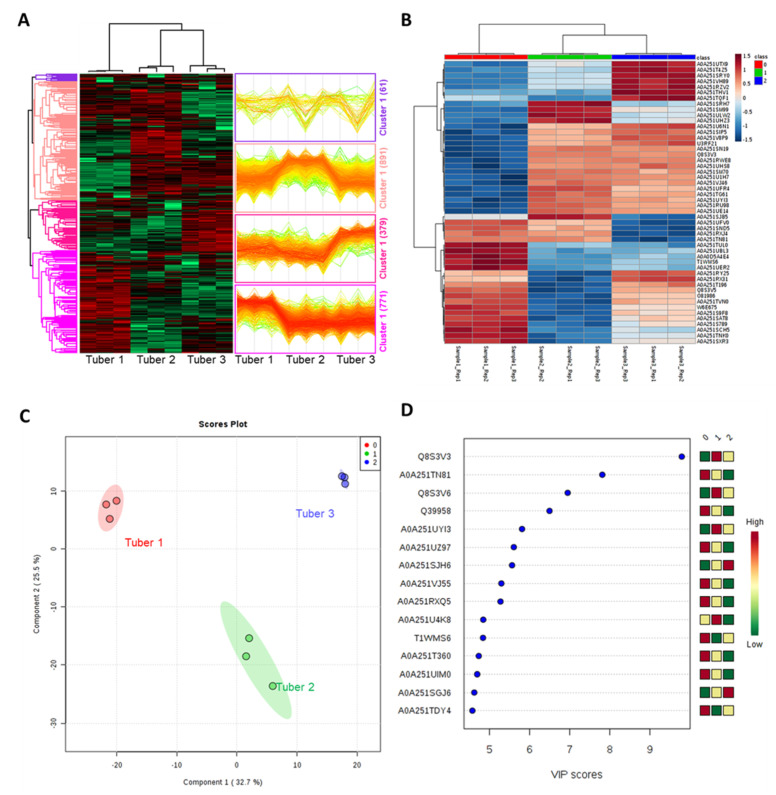
Statistical analysis of the JA tuber proteome. (**A**) Hierarchical clustering analysis on the total proteins from the three tubers showing differential proteins into four clusters, each with a distinct expression profile. (**B**) Heap map of top 48 differentially regulated proteins generated using MetaboAnalyst. Here, sample 1, 2, and 3 represent tubers 1, 2, and 3, respectively. (**C**) Principal component analysis of the differentially modulated proteins of tubers 1, 2, and 3. (**D**) Top 15 proteins with VIP values contributing to the separation in PLSDA plot. Here, in the color scale, 0, 1, 2 represent tubers 1, 2, and 3, respectively.

Hierarchical clustering analysis separated differential proteins into four clusters, each with a distinct expression profile (Figure 3A). Cluster 1 contained 61 proteins that are majorly downregulated in tuber 3, whereas 891 proteins of cluster 1 are mainly upregulated in tuber 2. Cluster 3 show 379 proteins with increased abundance in tuber 3 whereas 771 proteins of cluster 4 show maximum abundance in tuber 1. A multiple sample test controlled by a Benjamini–Hochberg FDR threshold of 0.01 was applied to identify the statistically significantly modulated proteins in the three tuber samples, resulting in the identification of 649 significant proteins (Supplementary Table S1, with + symbol in the ANOVA column). A heatmap showing the abundance pattern of the top 48 differentially modulated proteins is in (Figure 3B, Table 2). A variety of proteins with unique functions were identified in these 48 DEs. A total of 19, 24, and 34 DE proteins are highly modulated, whereas 27, 22, and 14 proteins are low in tubers 1–3, respectively. Results indicate that the protein content of tubers 1 and 3 are quite dissimilar, whereas tuber 2 exhibits a mixed result. Furthermore, the partial least squares-discriminant analysis (PLS-DA) separated tubers 1 and 3 in component 1, which accounts for 32.7% of the total variance, whereas tuber 2 was separated from tubers 1 and 3 in component 2, accounting for 25.5% of the total variance (Figure 3C). The top 15 proteins are identified which contribute to the separation of the PLS-DA plot (Figure 3D).

### 2.2. PANTHER-Based Proteome Categorization

**Figure 4:**
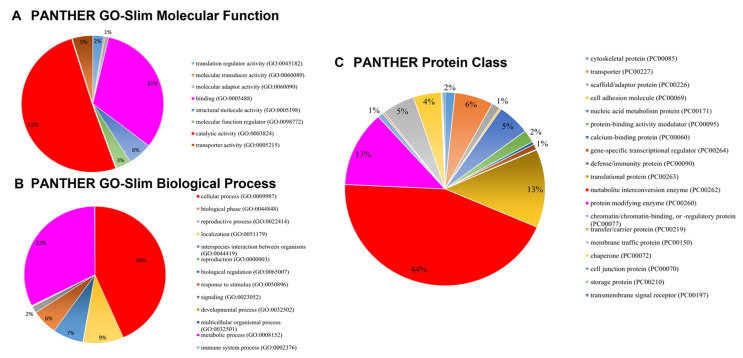
GO functional analysis and categorization of the JA tuber proteome in the current study using PANTHER tool. (**A**) Molecular function, (**B**) Biological process, (**C**) Protein class.

After statistical analysis, functional categorization based on gene ontology (GO) was performed to identify the organization of the tuber proteome. Out of 2102 proteins, 2044 proteins were detected by PANTHER 19.0 against the *Helianthus annus* database. Among these, 1457 protein hits were identified in eight categories of GO-molecular function (Figure 4A). The majority of the hits (51%) belong to catalytic activity (GO:0003824) and binding (GO:0005488, 32%). On the other hand, for the GO-biological process a total of 2119 hits are in 13 categories (Figure 4B). The majority of these proteins (44%) are found to be involved in the cellular process (GO:0009987), followed by the metabolic process (GO:0008152, 32%). Furthermore, the distribution of the proteome into various protein classes is also analyzed by the GO terms (Figure 4C). In this, 1523 hits were identified throughout 19 categories of protein classes. A large fraction of hits (44%) are found to be in the metabolite interconversion enzyme protein class (PC00262). The remaining proteome hits fall mostly in the translational protein (PC00263, 13%) and protein modifying enzyme (PC00260, 13%) categories.

**Figure 5:**
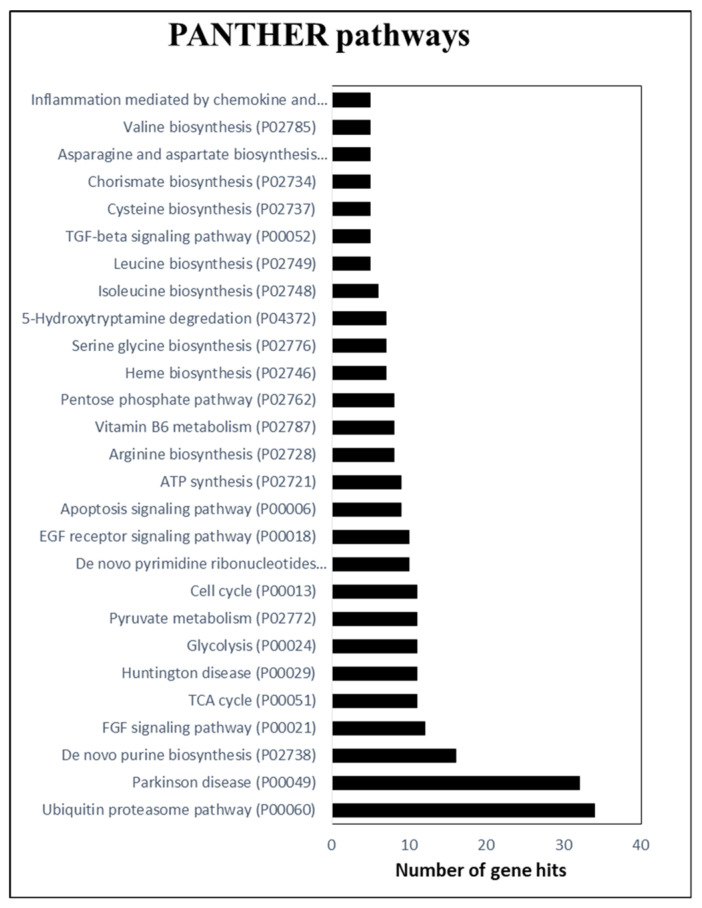
GO pathway categorization of the tuber proteome in the current study using PANTHER tool. Figure represents pathway categories to which 5 or more JA tuber gene hits were detected.

Next, the PANTHER pathway analysis was performed to categorize the protein pathways. In this, 411 gene hits are identified in 103 pathway categories (Supplementary Table S2). Figure 5 shows pathways in which five or more protein hits are detected. A majority of the protein hits are attributed to ubiquitin proteosome and nucleotide (purine) biosynthesis pathways, followed by TCA cycle, glycolysis, pyruvate metabolism, cell cycle, and pyrimidine ribonucleotide pathways.

## 3. Discussion

### 3.1. Selected Differentially Modulated Tuber Proteins with Unique Properties

Most of the differentially modulated proteins are found to have putative functions such as aldehyde dehydrogenase, polymyxin resistance, caffeoyl methyl transferase, cupredoxin, proteases, heat shock proteins (HSPs), peptidases, pyridoxin biosynthesis, etc. Very few of them were associated with specific functions. The protein “A0A0D5A4E4”, fructan 1-exohydrolase (1-FEH) is found in all the tubers and positively modulated in tuber 1 (Figure 3B, Table 2). Fructans such as inulin are abundant in JA tubers. These complex carbohydrates are essential dietary components that assist in improving healthy gut microbes as well as antidiabetic effect [45,46]. 1-FEH activity is associated with abiotic or biotic stress. It assists in the breakdown of fructans to provide carbohydrates and energy during stress responses [47,48]. This indicates that tuber 1 might be highly responsive to biotic or abiotic stress.

In addition to some starch biosynthesis pathway proteins, protein “O81986” a sucrose fructosyl transferase (1-SST) which is involved in inulin biosynthesis pathway is also identified. Recent studies suggest that 1-SST prioritizes inulin biosynthesis [27]. Results suggest that the two tubers 1 and 3 show high expression of 1-SST, and thus they could have high inulin synthesis potential (Figure 3B, Table 2). Besides its therapeutic applications, inulin is also considered as an essential ingredient of bioethanol and food supplements [49]. In relation to DB, another protein “A0A251S789”, prohibitin (PHB) is also found in the differentially modulated proteins of JA tubers (Figure 3B, Table 2). This protein is also highly modulated in tubers 1 and 3. PHBs are found in plants, fungi unicellular eukaryotes, and animals. In plants, it is involved in mitochondrial biogenesis [50]. In animals and humans, it regulates mitochondrial function. They have high therapeutic potential to treat age-related issues [51]. Studies show that increased levels of PHB1 are known to reduce the effects of DB2 [52,53], whereas some studies also show the potential of PHBs in cancer treatment [51,54].

Furthermore, we also identified peptide modification proteins. Three peptidylprolyl isomerase proteins “A0A251TN81”, “A0A251SCH5 (FKB12)”, and “A0A251UYI3 (ROF1)”, were also identified (Figure 3B, Table 2). These have different expression patterns in the three tubers. A0A251TN81 is highly modulated in tuber 1 and 2, A0A251UYI3 is highly modulated in tubers 2 and 3, and A0A251SCH5 in tuber 1 only and has low expression and vice versa. PPIases catalyse cis-trans isomerization of imide bonds between peptides and polypeptides [55,56]. These are found in all organisms. Arabidopsis thaliania is known to have a large number of PPIases compared with all other organisms [57]. In medical applications, some PPIases are targets for immune suppressant drugs such as cyclosporin A and FK506 [58,59]. Interestingly, the proteins “A0A251TN81” and “A0A251UYI3” are also found to contribute to PLS-DA separation of the three tubers with a high VIP score for tubers 1 and 2 (Figure 3C,D).

Another set of proteins “A0A251SRY0” and “A0A251VH89” which are related to phenylalanine ammonia-lyases (PAL) were also found (Figure 3B, Table 2). These two proteins are highly modulated in tuber 3, whereas in tubers 1 and 2 they have low expression. PAL enzymes are involved in the phenylpropanoid biosynthetic pathway in plants. Through this pathway PAL is responsible for production of cinnamic acid which is a precursor of several plant biomolecules such as flavonoids, hormones and linins. In JA tuber tissues, cinnamic acid-hydroxylyase (CAH) and PAL were found to have parallel activity changes [60]. PAL is also considered an essential therapeutic component to treat phenyketonuria (PKU). This is a condition where phenylalanine (Phe) cannot be hydroxylated to tyrosine and accumulated in the body tissues. Treating this condition with PAL helps in the conversion of Phe to benzoic acid and is renally excreted. Studies have shown that with oral administration of PAL produced from plants such as *Cyathobasis fruticulose (Bunge) Aellen. and Banana*, it is possible to treat phenylketonuria [61,62].

In addition to PAL, proteins that are found in organic compounds, and amino acids such as phenyl alanine, tyrosine and tryptophan were also identified. “A0A251UTX9” and “A0A251THV1” were identified as phospho-2-dehydro-3-deoxyheptonate aldolases that belong to the DAHP synthase family (Figure 3B, Table 2). These are involved in synthesis of volatile organic compounds and amino acids such as phenyl alanine, tyrosine and tryptophan in plants. These proteins were found to be highly modulated in tubers 3, and low in 1 and 2. S-adenosylmethionine synthases (SAMS1), “U3RF21” and “A0A251VJ46” were observed to be significantly modulated in tubers 2 and 3. SAMS1 proteins are previously reported [27]. All these enzymes are involved in amino acid synthesis in JA tubers. Additionally, a few more DE proteins which are involved in stress response and ion/water/metabolite transport proteins were also identified. Two tuber agglutinin proteins “Q8S3V3” and “Q8S3V5” were found to be highly modulated in tubers 1 and 3 and tubers 2 and 3, respectively. These are carbohydrate binding proteins which are involved in jasmonic-acid-induced plant defense mechanism during biotic and abiotic stress [63]. Among these two, “Q8S3V3” contributed to the PLS-DA separation of the three tubers (Figure 3C). From the PLS-DA analysis, another protein “Q39958” (aquaporin) was also found. This protein is also known to be modulated by animals. Aquaporins regulate water flux across the plasma membrane and regulates intestinal health [64]. Though this protein is helpful for plants, it is not suitable as a dietary nutrient as it may induce neuro autoimmune reactions [65]. According to PLS-DA, tubers 1 and 2 show high VIP scores for this protein. Therefore, tuber 3 could be a preferred dietary source. A0A251T360, a putative delta tonoplast integral protein was also found. These proteins are known to determine the function of the tonoplast such as aquaporins [66]. This protein also shows high VIP scores in tubers 1 and 2. Besides the biological significance of these proteins, such findings using PLS-DA in addition to DE proteins could indicate their role as biomarkers for specific tuber types as well.

### 3.2. Tuber Proteins Potentially Associated with Health and Disease Regulatory Metabolism

In the current study, through PANTHER pathway analysis we identified several proteins that were directly or indirectly involved in various disease-related mechanisms. One interesting finding is the identification of gene hits in pathways related to specific human disease pathways such as Alzheimer’s, Huntington, and Parkinson’s diseases (Figure 5, Supplementary Table S2). Three protein hits (A0A251ULZ4, A0A251V431, and A0A251VQN3) were identified to be associated with Alzheimer’s disease, among which “A0A251ULZ4” is listed in the 649 high-confidence proteins. All these belong to the mitogen-activated kinase (MAPK) family in JA tubers. MAPK proteins are found to be involved in biotic and abiotic stress signaling in plants [67]. The orthologue of A0A251ULZ4 in humans is found to be MAPK8 type known to be involved Alzheimer’s disease. A total of 11 protein hits were found to belong to the Huntington disease pathway, among which 6 proteins (A0A251T816, A0A251T3D2, A0A251RMC4, A0A251U0Y5, A0A251SQY8, and A0A251RRG5) were among most confidence proteins. The first 4 proteins are related to glyceraldehyde 3-phosphate dehydrogenases (GAPDH). In humans, GAPDH is known to form a complex with polyglutamine-expanded mutant huntingtin. Huntington disease is a neuro degenerative disease formed due to huntingtin protein structural deformation through the expansion of polyglutamine [68]. The orthologue of A0A251SQY8 in humans is an alpha-adaptin protein; this protein forms a complex with huntingtin and its interacting proteins, which are involved in clathrin mediated vesicle endocytosis [69]. Orthologue of A0A251RRG5 is a P53 tumor protein inducible protein PIG3. This PIG3 protein expression is affected by polyglutamine expanded mutant huntingtin protein in P53 dependent manner. In JA, these proteins belong to glyceraldehyde-3-phosphate dehydrogenases, oxidoreductases, and G-proteins. In JA, these proteins are involved in glycolysis, membrane trafficking, and redox reactions. In relation to Parkinson’s disease pathways, 32 protein hits were found. These proteins belong to proteases, dehydrogenases, adaptors, heat-shock-related proteins and threonine, and mitogen-activated protein kinases, among which “A0A251ULZ4”, “A0A251VFG3”, “A0A251UN40”, “A0A251TWQ3”, and “A0A251TU34” are in the 649 proteins. A0A251ULZ4, besides having orthologues related to Alzheimer’s disease, is also found to have orthologues related to stress-activated protein kinase (SAPK). However, the remainder, “A0A251VFG3”, “A0A251UN40”, and “A0A251TWQ3”, are heat shock proteins (HSP70). The HSP70 orthologues in humans are associated with parkin protein and the ubiquitination of Pale-R [70]. In JA and other plants, these HSPs are synthesized as a response to hear stress; although the orthologue of A0A251TU34 is a 20S proteosome, which is involved in degradation of ubiquitinated proteins such as parkin. In plants, these 20S proteosomes help fight against oxidative stress. These essentially play the same role of protein degradation, especially targeting oxidized proteins [71]. At present, the role of these plant-derived proteins in these human diseases is not well understood. Further studies could help us understand the significance of these plant-based proteins as therapeutic agents or dietary supplements to such disease conditions.

Apart from the above findings, we also identified proteins that are well known and have therapeutic significance, which is discussed in the following sub-sections. These are mostly useful in treating chronic diseases such as diabetes and cancer when consumed as a dietary protein source or through oral supplements. The majority of these proteins are associated with central carbon metabolism, vitamin metabolism, and diabetes-related proteins. Some antimicrobial proteins were also identified.

#### 3.2.1. Central Carbon Metabolism

Out of 2102 proteins, we identified 51 proteins associated with central carbon metabolism (Supplementary Table S1), out of which 22 proteins are among the 649 significant proteins; these protein numbers are indicated with parentheses. Among the 51(22) proteins, 3(2) are fructose-bisphosphate aldolase (FBA)-related proteins. In this notation, 3 is the total number of proteins identified among the 2102, and (2) indicates that 2 proteins are among the 649 significantly detected proteins. Similarly, 6(6) are glyceraldehyde-2-phosphate dehydrogenase (G3PDH)-related, 4(2) are enolase (ENO), 11(3) are pyruvate kinase (PK), 6(2) are pyruvate dehydrogenase (PDH), 8(0) are malate dehydrogenase (MDH), 5(4) are phosphoenolpyruvate carboxylase (PEPC), 3(0) are lipoamide dehydrogenase (LPD), and 5(3) are isocitrate dehydrogenase (IDH)-related proteins. All these proteins are essential for ATP synthesis, glycolysis, glucogenesis, plant growth, photo synthesis, and amino acid synthesis [27].

#### 3.2.2. Vitamins

Besides the core carbon metabolism proteins, we also identified proteins related to vitamin metabolism. A total of 9(2) proteins were identified as folic-acid-containing proteins, 8 proteins were identified in relation to pyridoxin (vitamin B6), 2(1) proteins were related to biotin, 1(1) protein was related to riboflavin, 2(0) proteins were related to thiamine, and 1(1) was related to vitamin D. Though some of these vitamin-related proteins are not in the significant protein list, the possibility of the presence of these vitamins cannot be ruled out. Most of these vitamins play a key role in treating type 2 diabetes mellitus [72]. Folic acid deficiency is known to be associated with several diseases such as cancer, cardiovascular disease, anemia, and type 2 DB. The supplementation of folic acid along with vitamins B6 and B12 could improve diabetic retinopathy [73]. The active form of vitamin B6 (pyridoxin) is pyridoxal-5′-phospate (PLP). This is commonly observed at low levels in diabetic patients. Studies showed that pyridoxamine supplementation decreases insulin concentration and sensitivity [74]. Among all the DE pyridoxine-related proteins found, we identified “T1WMS6” as a PLS-DA variable with a high VIP score for tuber 1, followed by tuber 3. Thiamine treatment increased renal clearance in both type 1 and type 2 DB patients [75] and also showed decreased glucose and leptin in diabetic patients [76]. In addition to these points, vitamin D, in its active form 1,25(OH)2D has a role in the regulation of the gene involved in insulin production and vascular smooth muscle cells. Studies suggest that vitamin D could play a key role in modifying the risk of diabetes and cardiovascular diseases [77,78]. Studies also showed that vitamin D also prevents free radical accumulation and thus can be an effective antioxidant [79].

#### 3.2.3. Diabetes-Related Proteins

Furthermore, we also found anti-diabetic-related proteins such as trehalose phosphorylase (TP), thaumatin, catalase (Q9M503, A0251U688, A0A251T1V1), profilin (A0A251U253), and glyceraldehyde-3-phosphate dehydrogenase (GADPH). Two TP-like proteins (2/0), “A0A251RS56” and “A0A251RNA0”, were identified in this study. Trehalose phosphorylase is an enzyme that mediates synthesis of trehalose sugars by degradation of *α*-glucose-1-phosphate (*α*-Glc-1-P) and glucose. Trehalose is a commercial sweetener used as a replacement of sucrose for diabetic patients. The presence of such enzyme indicates possible synthesis of trehalose in the tubers of JA. Studies also showed that uptake of trehalose reduce insulin resistance and osteoporosis development and maintains glycogen-trehalose balance in the body [80,81]. In contrast to trehalose such as sugars, we also identified a protein sweetener. This is a putative protein (A0A251SBX0) related to the thaumatin super family. Thaumatin is well known as a sweet protein and is mostly considered an alternate to artificial sweeteners as well as other natural carbohydrate-based sugars for diabetic patients [82].

Only one protein “A0A251U253” (1/0) was detected in relation to profilin. Profilin is found to have a high affinity for phosphatidylinositol 4,5-bisphosphate (PIP2); therefore, it could compete and inhibit the protein kinase C pathway. In the absence of profilin, the PKC pathway induces synthesis of diacyl glycerol and inositol triphosphate, leading to excessive calcium accumulation into the cell. This could lead to increased permeability and vascular cell proliferation, which in turn worsens the diabetic condition [83]. Profilin, on the other hand, prevents DM progression [84]. Furthermore, profilin is also considered as a biomarker for cancer as it plays a key role in actin assembly and microtubule dynamics [85]. In eukaryotes multiple isoforms of profilin exists. Studies showed that the endogenous over-expression of profilin 1 resulted in a tumor-suppressive nature [86,87,88]. However, the application of plant-based profilin as a therapeutic agent is yet to be studied. Three catalase-like proteins, Q9M503, A0A251U688, and A0A251T1V1, were identified. Out of these, “Q9M503” was identified to be in the significant protein list. This enzyme is involved in the breakdown of hydrogen peroxide, and enhances insulin secretion and sensitization. Catalase deficiency may cause hydrogen-peroxide-dependent oxidative stress and thus damage to pancreas and insulin signaling [89]. The consumption of food products that consist of catalase could also improve blood catalase levels and thus prevent diabetic conditions.

For GADPH, we identified seven proteins, out of which five are found in the highly significant protein list. In diabetic conditions, GAPDH activity is downregulated due to limited nucleotide availability [90]. This results in the formation of methylglyoxal (MG) and advanced glycation end products (AGEs) which induce oxidative stress (OS), leading to vascular complications and mitochondrial dysfunction [91,92]. Therefore, regulating GADPH levels could help prevent mitochondrial dysfunction. However, the significance of GAPDH as a dietary component is not well known.

In addition, the current study also found proteins related to antioxidants that could help ameliorate AGE-related OS. These proteins (A0A251VP43, A0A251UYL3, A0A251UKV9, A0A251T1K2, and A0A251V8Z5) NAC-A/B containing domain and n-acetyl transferases are part of n-acetyl cystine (NAC) metabolism. NAC play key roles in free radical scavenging and in improving glutathione levels (Likapolus et al., 2019). Three phosphoglucomutase (PGMP) family proteins (A0A251SKR1, A0A251TSC3, and A0A251S377 3/0) were also identified here. PGMP participates in glucose catabolism and anabolism [93]. Feeding diabetic rats with *Chrysobalanus icaco* fruits leaves and fruits consisting of the PGMP protein showed decreases in blood sugar levels [94].

#### 3.2.4. Other Antimicrobial Proteins

The tuber proteome also revealed antimicrobial-related proteins which also have therapeutic applications. Two proteins (A0A251U2V7 and A0A251UER2) related to Kunitz-type protease inhibitors were also detected. These are known to have antifungal and anticancer activities [95,96]. In addition, we also identified proteins related to Serine hydroxyl methyl transferases (SHM1), which are considered therapeutic targets for antimicrobial and antineoplastic agents [97].

## 4. Materials and Methods

### 4.1. Specification of Samples and Total Protein Extraction

The JA tuber samples, as commercial products (shown in Supplementary Figure S1—bottom left, and marked as 1, 2, and 3) used for consumption, were obtained from local agricultural communities as follows: (1) JA tuber powder from Oguni town in Kumamoto prefecture (the tubers were placed in a drying hut for local agricultural products where steam generated through a hot spring was piped to complete the natural drying process and then powdered); (2) JA tuber powder from Shima city, Mie prefecture (the sliced tuber was ordinarily warm-air dried and then powdered); and, (3) JA tuber also from Shima city (Mie prefecture) was sliced and sun-dried to produce the JA tuber chips. For protein extraction, 1 g of dried tuber powder (and finely powdered in liquid nitrogen) from three tuber samples was homogenized in 10 mL of Tris-Mg/NP-40 buffer (0.5 M Tris-HCl (pH 8.3), 2% (*v*/*v*) NP-40, 20 mM MgCl2) followed by centrifugation at 12,000× *g* for 15 min at 4 °C. Supernatant so obtained was subjected to methanol-chloroform precipitation method twice followed by final washing of pellet with 80% acetone containing 0.07% β-mercaptoethanol.

### 4.2. Sample Preparation and LC-MS/MS for Proteome Analysis

For proteome analysis, protein pellets after methanol-chloroform precipitation were dissolved in the SDT-lysis buffer containing 4% SDS, 100 mM Tris/HCl pH 7.6 and 0.1 M DTT. After sonication for 1 min, samples were incubated at 95 °C for 30 min after which these were allowed to cool at room temperature for 15 min and protein concentration in each sample was measured by 2D-Quant kit (GE Healthcare) following manufacturer’s protocol. A total of 100 µg of proteins from each sample was used for trypsin digestion by filter-aided sample preparation (FASP) method as described earlier [98] and peptides so obtained were quantified using PierceTM Quantitative Fluorometric Peptide Assay (Thermo Scientific, Waltham, MA, USA) following manufacturer’s protocol. Peptides, thus obtained, were desalted using Oasis^®^ HLB 1cc (360 mg) solid-phase extraction (SPE) cartridge (Waters, Milford, MA, USA) following manufacturer’s instructions, and the final eluate was lyophilized.

### 4.3. Q-Exactive MS Analysis

Lyophilized peptides were dissolved again in solvent-A (water/ACN, 98:2 *v*/*v*; 0.1% formic acid) and separated by reversed-phase chromatography using a UHPLC Dionex UltiMate^®^ 3000 (Thermo Fisher Scientific, Waltham, MA, USA) instrument [99]. For trapping the sample, the UHPLC was equipped with Acclaim PepMap 100 trap column (100 μm × 2 cm, nanoViper C18, 5 μm, 100 Å) and subsequently washed with 98% solvent A for 6 min at a flow rate of 6 μL/min. The sample was continuously separated on an Acclaim PepMap 100 capillary column (75 μm × 15 cm, nanoViper C18, 3 μm, 100 Å) at a flow rate of 400 nL/min. The LC analytical gradient was run at 2% to 35% solvent B (100% ACN and 0.1% formic acid) over 90 min, then 35% to 95% over 10 min, followed by 90% solvent B for 5 min, and finally 5% solvent B for 15 min. Liquid chromatography-tandem mass spectrometry (LC-MS/MS) was coupled with an electrospray ionization source to the quadrupole-based mass spectrometer QExactive™ Orbitrap High-Resolution Mass Spectrometer (Thermo Fisher Scientific, Waltham, MA, USA). Resulting peptides were electro-sprayed through a coated silica emitted tip (Scientific Instrument Service, Amwell Township, NJ, USA) at an ion spray voltage of 2000 eV. The MS spectra were acquired at a resolution of 70,000 (200 *m*/*z*) in a mass range of 350–1650 *m*/*z*. The automatic gain control (AGC) target value was 3 × 106 and the isolation window for MS/MS was 1.2 *m*/*z*. Eluted samples were used for MS/MS events (resolution of 35,000), measured in a data-dependent mode for the 15 most abundant peaks (Top15 method), in the high mass accuracy Orbitrap after ion activation/dissociation with Higher Energy C-trap Dissociation (HCD) at 32 collision energy in a 100–1650 *m*/*z* mass range. The maximum ion injection time for the survey scan and MS/MS scan was 30 ms and 120 ms, respectively [100].

### 4.4. LC-MS/MS Data Analysis for Proteome Comparisons

The acquired MS data were analyzed with MaxQuant (ver. 1.5.3.30) [101]. MS/MS spectra were searched with the integrated Andromeda search engine against the Helianthus protein database downloaded from UniProt and 248 common contaminant proteins. Trypsin specificity was required and a maximum of two missed cleavages allowed. Minimal peptide length was set to seven amino acids. Carbamidomethylation of cysteine residues was set as fixed, oxidation of methionine and protein N-terminal acetylation as variable modifications. Allowed mass deviation was 4.5 ppm for peptides and 20 ppm for fragments. Label-free quantification (LFQ) data were processed using the default precursor mass tolerances set by Andromeda with mass deviation was 4.5 ppm for peptides and 20 ppm for fragments with enabled stabilization of large LFQ ratios and LFQ normalization study [102]. The LFQ data were searched based on 0.5 Da of a product mass tolerance with a maximum of two missed cleavages allowed. Minimal peptide length was set to seven amino acids. Peptide-spectrum matches and proteins were retained if they were below a false discovery rate of 1%. Statistical analyses were carried out using Perseus software (ver. 1.5.8.5) [103]. Hits were only retained if they were quantified in at least two of the three replicates in at least one experiment. The missing value imputation of protein intensities was performed from a normal distribution (width: 0.3, down shift: 1.8). Multiple sample test (ANOVA), controlled by the Benjamini–Hochberg FDR threshold of 0.01, was applied to identify significant differences in the protein abundance. Functional annotation of the identified proteins was carried out by PANTHER and Gene Ontology tools. Furthermore, partial least squares discriminant analysis (PLS-DA) plots were generated by the MetaboAnalyst online tool [104].

### 4.5. Mass Spectrometry Protein Data Access

The mass spectrometry proteomics data have been deposited to the ProteomeXchange Consortium via the PRIDE [105,106,107] partner repository with the dataset identifier PXD030744.

## 5. Conclusions

*Jerusalem artichoke* tuber proteomes from two different processing techniques, dry powder (tuber 1 and 2), and dry chips (tuber 3), were unraveled using a high-throughput label-free LC-MS/MS-based omics technology. Out of the 3065 proteins detected, only 2967 were identified with high confidence. Among the many different proteins identified relating to health and disease, our data particularly revealed the presence of 1-SST, which is involved in inulin biosynthesis. This was a main reason that our group chose to study this plant. The proteins identified, and their classifications, suggest functions which support data (as yet unpublished) on kiku-imo: data which suggest a reduction effect on blood sugar levels, and a relation to glycated hemoglobin (Hb-A1c) in humans (Genboku Takahashi et al., n.d., unpublished data). These protein data constitute a novel experimental dataset for the tuber samples, which are a part of the healthy diet and lifestyle of Japanese people. We hope that this research will lead to both a renewed interest in the study of the ‘kiku-imo’ at the clinical stages, as well as promote the use of omics technologies, including proteomics, to identify novel components of the ‘healthy’ foods in our diets. The next step of the research which was not examined here is to further understand how the obtained differences in proteins among different samples relate to the processing steps and how each may be beneficial to human health. These data may also be related to the tubers harvested in different regions within Japan.

## Figures and Tables

**Table 1:** Total number of proteins in each sample along with their replication ratio, coefficient of variation percentage, and standard deviation.

Sample	Rep_1	Rep_2	Rep_3	Common Proteins in All Replicates	Standard Deviation	Replication Ratio	Percentage Coefficient of Variation (CV%)
Sample 1	1917	1966	1955	1780	25.71	0.915	8.5
Sample 2	1901	1898	1920	1732	11.93	0.909	9.1
Sample 3	1887	1917	2439	1701	310.4	0.817	18.26

**Table 2:** The identified top 48 *Helianthus tuberosus* L. (Kiku-imo) tuber proteins.

S.No	Protein IDs	Annotation	No of Peptides
1	A0A251UTX9	Phospho-2-dehydro-3-deoxyheptonate aldolase	12
2	A0A251T4Z5	Putative caffeoyl-CoA O-methyltransferase	8
3	A0A251SRY0	Phenylalanine ammonia-lyase	16
4	A0A251VH89	Phenylalanine ammonia-lyase	17
5	A0A251RZV2	Putative bifunctional polymyxin resistance protein, ArnA	6
6	A0A251THV1	Phospho-2-dehydro-3-deoxyheptonate aldolase	13
7	A0A251TQF1	Putative FAD-binding Berberine family protein; Belongs to the oxygen-dependent FAD-linked oxidoreductase family.	6
8	A0A251SRH7	Putative cobalamin-independent methionine synthase	45
9	A0A251SM99	Putative heat shock protein Hsp90 family	31
10	A0A251ULW2	Putative glucose/ribitol dehydrogenase; Belongs to the short-chain dehydrogenases/reductases (SDR) family.	6
11	A0A251UHZ3	Glutathione peroxidase; Belongs to the glutathione peroxidase family.	5
12	A0A251U6N1	Putative caffeic acid 3-O-methyltransferase; Belongs to the class I-like SAM-binding methyltransferase superfamily. Cation-independent O-methyltransferase family.	9
13	A0A251SIP5	UDP-glucose 6-dehydrogenase	13
14	A0A251V8P9	Putative triose phosphate/phosphoenolpyruvate translocator	2
15	U3RF21	S-adenosylmethionine synthase	16
16	A0A251SN19	Peroxidase; Removal of H(2)O(2), oxidation of toxic reductants, biosynthesis and degradation of lignin, suberization, auxin catabolism, response to environmental stresses such as wounding, pathogen attack and oxidative stress.	7
17	Q8S3V3	Tuber agglutinin	12
18	A0A251RWE8	Putative heat shock protein; Belongs to the ClpA/ClpB family.	35
19	A0A251UHS8	Methylenetetrahydrofolate reductase; Belongs to the methylenetetrahydrofolate reductase family.	12
20	A0A251SM70	Putative pyridoxine biosynthesis 1.2; Belongs to the PdxS/SNZ family.	6
21	A0A251U1H7	Putative HSP20-like chaperone; Belongs to the small heat shock protein (HSP20) family.	4
22	A0A251VJ46	S-adenosylmethionine synthase; Catalyzes the formation of S-adenosylmethionine from methionine and ATP.	12
23	A0A251UFR4	Putative 22.0 kDa class IV heat shock protein; Belongs to the small heat shock protein (HSP20) family.	7
24	A0A251TG61	Peroxidase; Removal of H(2)O(2), oxidation of toxic reductants, biosynthesis and degradation of lignin, suberization, auxin catabolism, response to environmental stresses such as wounding, pathogen attack and oxidative stress.	10
25	A0A251UYI3	Peptidylprolyl isomerase	9
26	A0A251RU98	Putative HSP20-like chaperone; Belongs to the small heat shock protein (HSP20) family.	7
27	A0A251UE14	Putative casein lytic proteinase B3; Belongs to the ClpA/ClpB family.	23
28	A0A251SJB5	Putative sieve element occlusion	10
29	A0A251UFV0	Putative P-loop containing nucleoside triphosphate hydrolases superfamily protein	16
30	A0A251SND5	Putative RNA-binding (RRM/RBD/RNP motifs) family protein	6
31	A0A251RXJ4	SUMO-activating enzyme subunit; Belongs to the ubiquitin-activating E1 family.	4
32	A0A251TN81	Peptidyl-prolyl cis-trans isomerase; PPIases accelerate the folding of proteins. It catalyzes the cis-trans isomerization of proline imidic peptide bonds in oligopeptides; Belongs to the cyclophilin-type PPIase family.	6
33	A0A251TUL0	Putative peptidase C13, legumain	4
34	A0A251UBL3	Putative START-like domain-containing protein	8
35	A0A0D5A4E4	Fructan 1-exohydrolase	6
36	T1WMS6	GLYCENE RICH RNA BINING PROTEIN	4
37	A0A251UER2	Putative alpha-amylase/subtilisin inhibitor	5
38	A0A251RY25	Putative granulin; Belongs to the peptidase C1 family.	4
39	A0A251RX31	Putative aldehyde dehydrogenase 2B4; Belongs to the aldehyde dehydrogenase family.	19
40	A0A251T196	Putative cupredoxin	4
41	Q8S3V5	TUBER AGGULTIN	8
42	O81986	SUCROSE FRUCTOSYL TRASFERASE	12
43	A0A251S9F8	Putative eukaryotic aspartyl protease family protein; Belongs to the peptidase A1 family.	4
44	A0A251SAT8	UTP--glucose-1-phosphate uridylyltransferase	22
45	A0A251S789	Prohibitin	11
46	A0A251SCH5	Peptidylprolyl isomerase	3
47	A0A251TNK0	Putative cytochrome P450; Belongs to the cytochrome P450 family.	6
48	A0A251SXR3	Putative oxysterol-binding family protein; Belongs to the OSBP family.	5

## Data Availability

All raw data will be shared as requested.

## Supplementary Materials

The following supporting information can be downloaded online. Figure S1: Kiku-imo plant growth stages from sprouting to tuber formation and harvesting; Table S1: Curated proteome data containing 2102 proteins; Table S2: Table representing PANTHER pathways identified for the proteins.

## Sample Availability

Samples of the compounds are not available from the authors.
