# Supplementary material for: Unravelling the Helianthus tuberosus L. (Jerusalem Artichoke, Kiku-Imo) Tuber Proteome by Label-Free Quantitative Proteomics
Source: Molecules. 2022 Feb 7;27(3):1111. doi: 10.3390/molecules27031111 (PMC8840128; doi:10.3390/molecules27031111)
Supplement: Supplementary file 1 [file molecules-27-01111-s001.zip › Supplementary Figure S1.pdf]

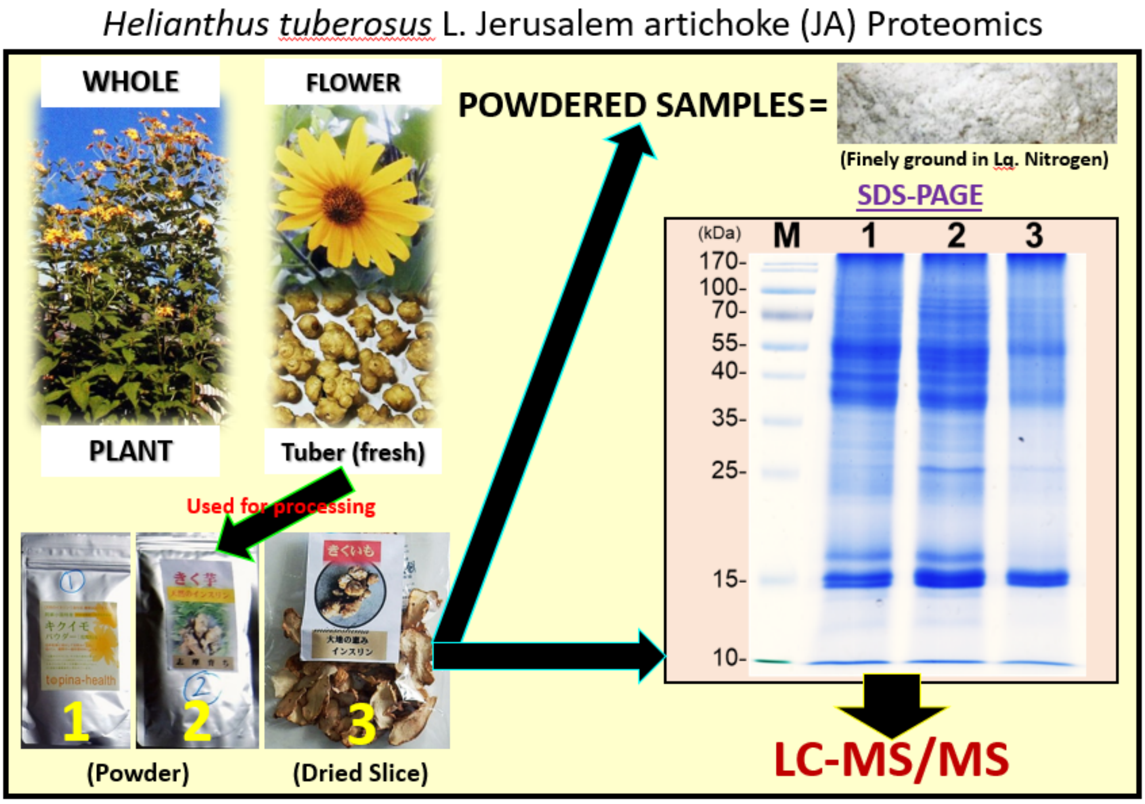

Supplementary Figure S1. Workflow for the proteome identification of the 'Kiku-imo' (*Helianthus tuberosus* L.) commercial edible samples. The upper left images are of the plant, flowers and the tubers. Below, the 3 commercial samples used (as also described in Materials and Methods), 1 and 2 are dried powders, and the 3 is dried chips.. All samples were finely ground in liquid nitrogen and the extracted protein pattern was examined by SDS-PAGE and CBB staining to confirm clear protein patterns and no protein degradation, before proceeding to the main MS (LC-MS/MS) step (Materials and Methods).
